# Autodisplay for the co-expression of lipase and foldase on the surface of *E. coli*: washing with designer bugs

**DOI:** 10.1186/1475-2859-13-19

**Published:** 2014-01-29

**Authors:** Eva Kranen, Christian Detzel, Thomas Weber, Joachim Jose

**Affiliations:** 1 Institute of Pharmaceutical and Medicinal Chemistry, PharmaCampus, Westfalian Wilhelms-University Münster, Corrensstr. 48, 48149 Münster, Germany; 2 Autodisplay Biotech GmbH, Merowingerplatz 1a, Düsseldorf, 40225, Germany; 3 International R&D/Technology Laundry & Homecare, Henkel AG & Co. KGaA, Düsseldorf, 40191, Germany

## Abstract

**Background:**

Lipases including the lipase from *Burkholderia cepacia* are in a main focus in biotechnology research since many years because of their manifold possibilities for application in industrial processes. The application of *Burkholderia cepacia* lipase for these processes appears complicated because of the need for support by a chaperone, the lipase specific foldase. Purification and reconstitution protocols therefore interfere with an economic implementation of such enzymes in industry. Autodisplay is a convenient method to express a variety of passenger proteins on the surface of *E. coli*. This method makes subsequent purification steps to obtain the protein of interest unnecessary. If enzymes are used as passengers, the corresponding cells can simply be applied as whole cell biocatalysts. Furthermore, enzymes surface displayed in this manner often acquire stabilization by anchoring within the outer membrane of *E. coli*.

**Results:**

The lipase and its chaperone foldase from *B. cepacia* were co-expressed on the surface of *E. coli* via autodisplay. The whole cell biocatalyst obtained thereby exhibited an enzymatic activity of 2.73 mU mL^-1^ towards the substrate *p*-nitrophenyl palmitate when applied in an OD_578_ =1. Outer membrane fractions prepared from the same culture volume showed a lipase activity of 4.01 mU mL^-1^. The lipase-whole cell biocatalyst as well as outer membrane preparations thereof were used in a standardized laundry test, usually adopted to determine the power of washing agents. In this test, the lipase whole cell biocatalyst and the membrane preparation derived thereof exhibited the same lipolytic activity as the purified lipase from *B. cepacia* and a lipase preparation which is already applied in commercial washing agents.

**Conclusions:**

Co-expression of both the lipase and its chaperone foldase on the surface of *E. coli* yields a lipid degrading whole cell biocatalyst. Therefore the chaperone supported folding process, absolutely required for the lipolytic activity appears not to be hindered by surface display. Furthermore, the cells and the membrane preparations appeared to be stable enough to endure a European standard laundry test and show efficient fat removal properties herein.

## Background

Lipolytic enzymes are attractive biotechnological tools [1]. Among them lipases (triacylglycerol acylhydrolases EC 3.1.1.3), which catalyze the hydrolysis of triglycerides in aqueous media, liberating free fatty acids and glycerol, or the reverse reaction in organic solvents as well, have gained particular interest, since they simultaneously show high enantio- and/or regio-selectivity as well as a high catalytic activity and thermostability in organic solvents [2,3]. Contrary to esterases, which preferentially break ester bonds of short chain fatty acids, lipases are able to catalyze the hydrolysis of water-insoluble long-chain acylglycerols [1]. Interestingly, activation of lipases often depends on the presence of a lipid-water interface, which can be explained by their three-dimensional structure. In an enzymatically inactive state, a surface loop, the so-called lid, covers the active site of the lipase. Upon contacting the lipid-water interface the lid switches open, and the active site becomes accessible for the substrate [4].

So far, lipases have been established in numerous industries, such as the food industry, paper manufacturing, pharmaceutical processing [5], and detergents industry, reflecting their great importance [4]. Despite this enormous industrial interest, not more than around 20 lipases have been established for industrial applications yet [6]. The sometimes troublesome and time-consuming purification procedures to obtain pure enzyme preparations for particular applications seem to be one possible obstacle in broadening the use of lipases in industrial processes [7]. Moreover, to express lipases from *Burkholderia* and *Pseudomonas* species in an active form, lipases which have advantageous features regarding thermal stability, alkaline pH tolerance and high substrate selectivity, and therefore making them promising industrial biocatalysts [8-10], bears an additional problem. These enzymes are dependent on the presence of a personal chaperon, the so-called lipase-specific foldase (Lif), responsible for correct folding of the lipase [1,11]. As a consequence, former heterologous expression of the *Burkholderia cepacia* lipase in *E. coli* resulted in a very low yield of active soluble lipase, whereas the majority of the enzyme was expressed as insoluble inclusion bodies. Significant amounts of active lipase were only achieved by applying an additional in-vitro refolding protocol [12].

An innovative way to gain access to the synthetic potential of lipases is their display on the surface of a living cell, in particular an *E. coli* cell [13]. Since the enzyme is directly accessible for its substrate, costly purifications as mentioned above are not necessary.

**Figure 1:**
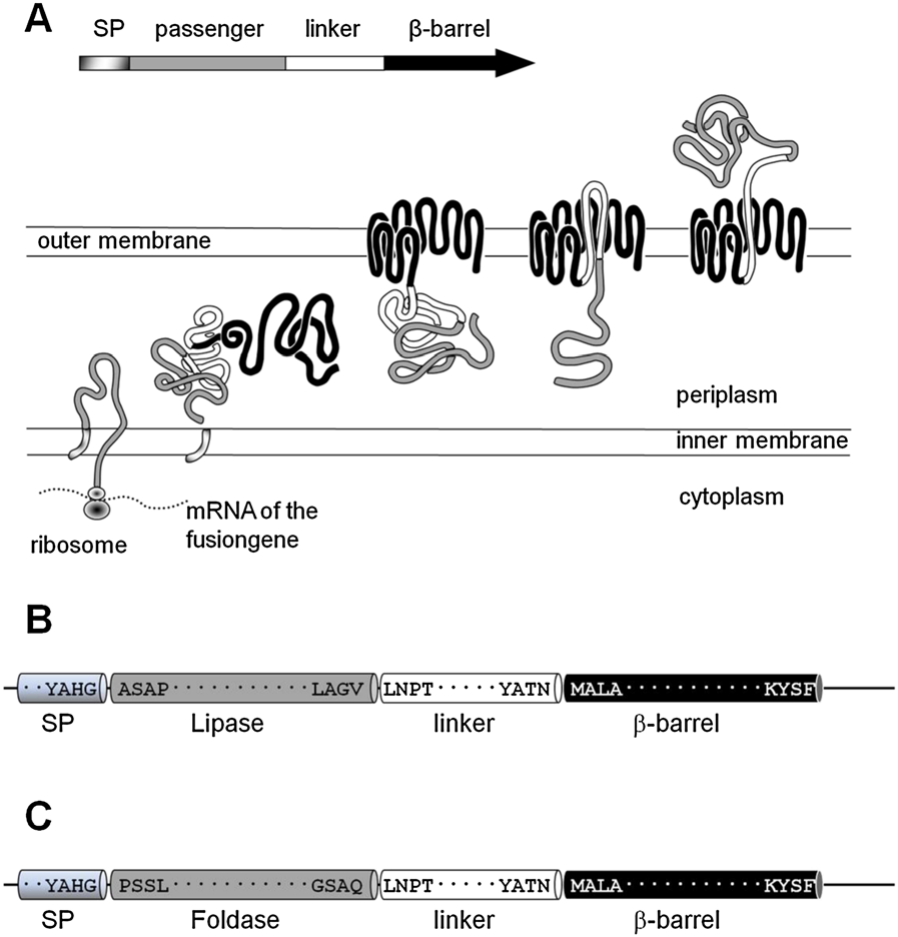
**Passenger transport across two membranes by Autodisplay. A**: The N-terminal signalpeptide facilitates the transport across the inner membrane by the so called Sec-pathway [32] and is then cut off by periplasmic signal peptidases. The C-terminal part forms a porin-like β-barrel structure inside the outer membrane through which the passenger is translocated to the surface by the linker. **B** and **C**: The structure of the precursor proteins for LipBC-FP **(B)** and FoldBc-FP **(C)** is shown schematically. The mature fusion proteins anchored inside the outer membrane only consist of the passenger (which would be here lipase or foldase, depicted in lightgrey) and the autotransporter structure (linker (white) and β-barrel (black)). (The genes for lipase and foldase were amplified from plasmid pHES8, containing the complete sequence of the *B. cepacia lipase* [GenBank:FJ638612] and cloned into appropriate Autodisplay-vectors via restriction sites XhoI (5′-end) and KpnI (3′-end).

So far, various anchoring motifs like OmpC [14], ice nucleation protein [15], OprF [16] and FadL [17] have been used to display *Pseudomonas* and *Bacillus* lipases on the surface of *E. coli.* Wilhelm *et al.*[18] were able to display the LipH chaperone of *P. aeruginosa* in an active state on the surface of *E. coli* by using the *P. aeruginosa* autotransporter protein EstA. With these cells displaying the lipase specific foldase, reconstitution of a purified but denatured lipase into an active form was facilitated. In another report, Yang *et al.* described the display of active *P. aeruginosa* and *B. cepacia* lipases on the surface of *E. coli* via co-expression of lipase and the Lif protein within a single fusion protein [19]. Autodisplay, a bacterial surface display system, appeared to be a convenient tool for the expression of *B. cepacia* lipase, since it has been proven to be well adapted for the surface display of challenging enzymes. As an example it was possible to express enzymatically active human hyaluronidases in *E. coli*, a group of enzymes which are known to form inclusion bodies, when expressed by other means [20]. Autodisplay is based on AIDA-I, the adhesin involved in diffuse adherence in enteropathogenic *E. coli* (EPEC) [21,22], a naturally occurring autotransporter protein in *E. coli*. The gene construct applied in Autodisplay encodes a fusion protein comprised of an N-terminal signal peptide derived from cholera toxin β-subunit (CtxB), a variable passenger domain and the C-terminal AIDA-I autotransporter including a linker to enable full surface access of the passenger domain (Figure 1B). Most probably, the linker and the β-barrel are responsible for the translocation of the passenger protein across the *E. coli* outer membrane (Figure 1A). One of the most striking features of the Autodisplay system is the mobility of the β-barrel serving as an anchor within the outer membrane. This enables the self-driven dimerization or multimerization of subunits to active or functional enzymes on the surface of *E. coli,* even in case they were expressed as monomers*.* Examples for this self-driven dimerization or multimerization of passsenger proteins on the cell surface of *E. coli* are the active display of dimeric adrenodoxin [23], dimeric sorbit dehydrogenase [24], multimeric nitrilase [25] and dimeric prenyl transferase [26]. Moreover, Autodisplay has proven to be a robust expression platform for the surface display of enzymes in general including cytochrome P450 enzymes of bacterial and human origin [27-29]. More recently, it was shown that Autodisplay, which is defined as the surface display of a recombinant protein by the autotransporter secretion pathway [30], relies on a set of periplasmic chaperones including a complex of proteins which corresponds to the so-called Bam machinery in *E. coli*[31]. This makes the prefix “auto” somewhat obsolete, but for clarity reasons it appears to be favorable not to change the term Autodisplay on these findings. In order to elucidate, whether Autodisplay is not only capable of permitting subunits of enzymes to aggregate on the cell surface, but can also be used for the expression of two different enzymes on a single cell, we chose *Burkholderia cepacia* lipase and its specific foldase as candidates. Lipolytic activity was tested in common lab scale assays as well as in a standardized laundry test which is typically used to evaluate the quality of washing agents. Since the presence of recombinant bacteria in clothes after washing could cause some resistance in application, also membrane preparations of the cells co-expressing lipase and foldase were applied in the identical test as well.

## Results

### Construction of the plasmid for autodisplay of lipase

By analyzing the amino acid sequence of *B. cepacia* ATCC 21808 lipase using the SignalP computer program [33], a classical signal peptide was identified at its N terminus. Since this lipase inherent signal peptide is proposed to interfere with the signal peptide used in autodisplay and thus constrain a proper transport across the inner membrane, the lipase signal peptide encoding 120 bp sequence was deleted by PCR. PCR-primers were designed according to the deposited sequence of the *B. cepacia* lipase [GenBank: FJ638612] and added an XhoI (5′end) and a KpnI restriction site (3′end) to the PCR fragment in order to enable an in frame fusion with the plasmid DNA encoding the autodisplay domains. For PCR plasmid pHES8 was used, which resembles pHES12 described by Quyen et al. [12] and encodes the complete *B. cepacia* lipase operon (i.e. lipase and its corresponding foldase) for intracellular expression in *E. coli*. After insertion into plasmid pCD003 [25] cleaved with XhoI and KpnI as well, plasmid pAT-LipBc was obtained encoding a fusion protein comprising the signal peptide of CtxB at the N terminus followed by the lipase as a passenger, the linker region and the β-barrel from the AIDA-I autotransporter needed for outer membrane translocation and full surface accessibility (Figure 1B).

### Surface display of lipase

**Figure 2:**
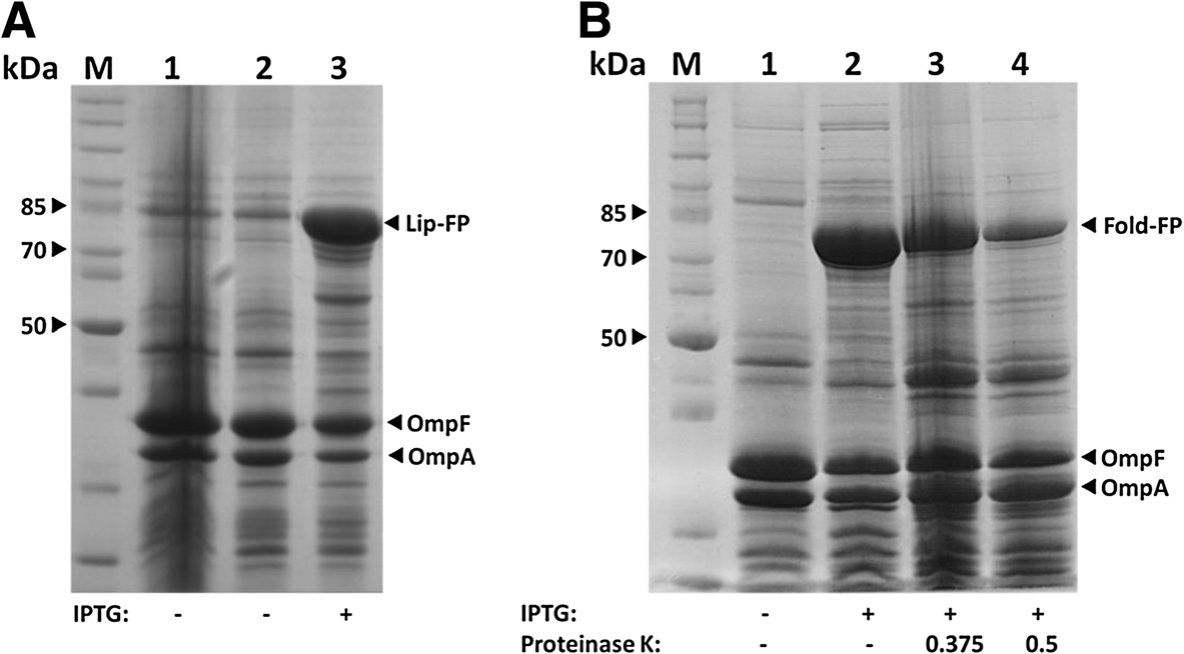
**Expression of lipase fusion protein, expression and surface display of foldase fusion protein. A**: SDS-PAGE of the outer membrane protein preparation of *E. coli* BL21(DE3)pAT-LipBc. Lane 1 shows an outer membrane preparation of *E. coli* BL21(DE3), used as a control. Lanes 2 and 3 show outer membrane preparations of *E. coli* BL21(DE3)pAT-LipBc. **B**: SDS-PAGE of the outer membrane protein preparation of *E. coli* BL21(DE3)pAT-FoldBc. Molecular weight markers are indicated on the left hand side. M: protein marker; IPTG: protein expression was induced by adding IPTG (final concentration: 1 mM); Proteinase K: whole cells were treated with Proteinase K; concentrations are given in mg mL^-1^. The lipase and foldase fusion proteins are indicated by using black arrows. OmpA/OmpF: native *E. coli* outer membrane proteins are also indicated by a black arrow.

**Figure 3:**
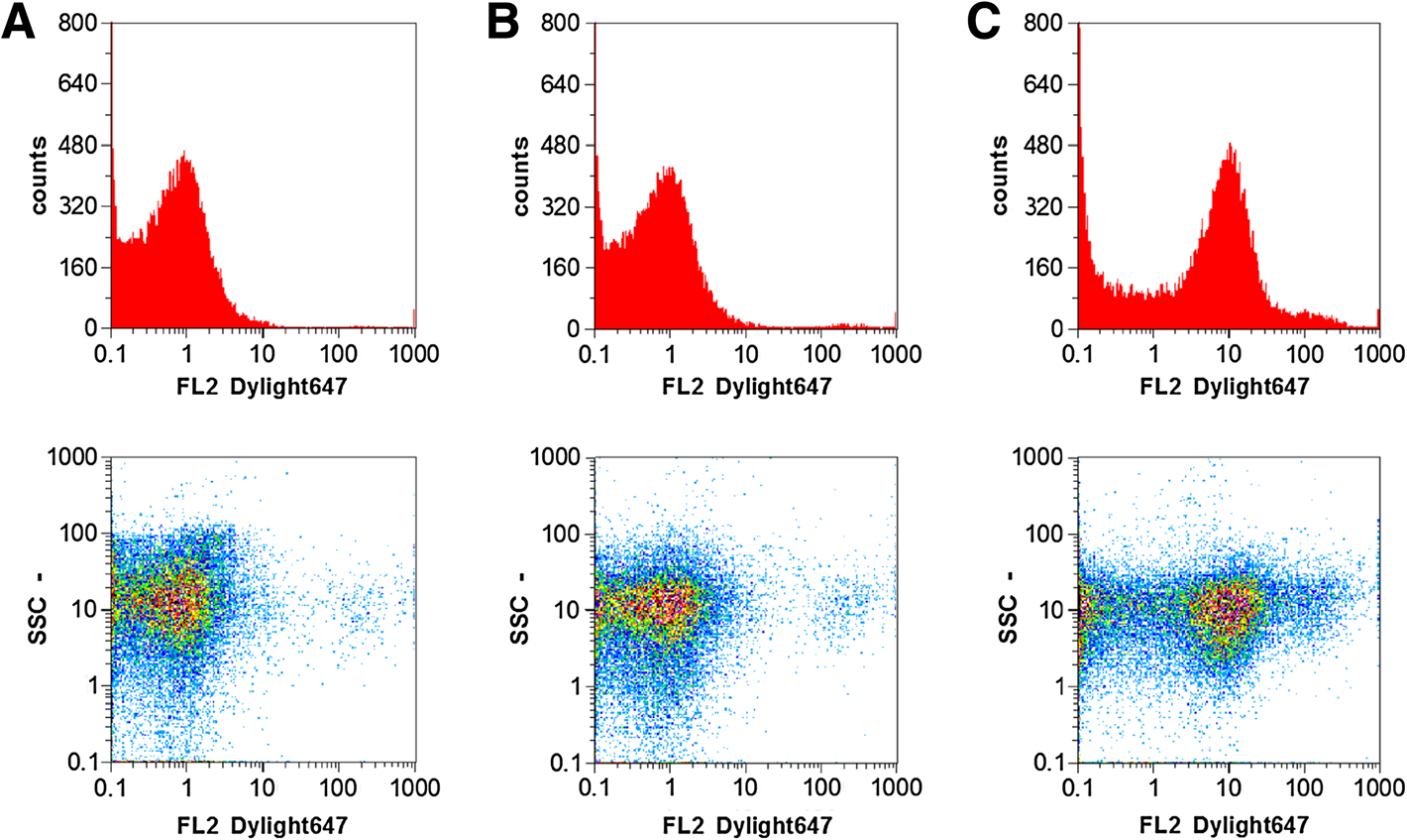
**FACS analysis of lipase surface display.** Whole cells were treated with rabbit-anti-lipase-antibody and anti-rabbit Dylight coupled secondary antibody. **A**: host cells *E. coli* BL21(DE3) used as a control, **B**: Cells displaying another enzyme on their surface (*E. coli* BL21(DE3)pAT-NOx). These were used as control cells to test whether the anti-lipase-antibody binds parts of the autotransporter. **C**: BL21(DE3)pAT-LipBc expressing the lipase via Autodisplay. Only the reaction with BL21(DE3)pAT-LipBc and the lipase-antibody caused a tenfold increase in mean fluorescence values, which means that the lipase can be regarded as surface exposed.

*E. coli* BL21(DE3) pAT-LipBc were grown until an OD_578_ of 0.5 was reached. Expression of the lipase fusion protein was then induced by addition of isopropyl-β-thiogalactosid (IPTG) to a final concentration of 1 mM and incubation for one hour. Adjacently cells were harvested and the outer membrane proteins were isolated according to the protocol of Hantke [34], modified by Schultheiss *et al*. [35]. The obtained outer membrane preparations were then subjected to SDS-PAGE to analyze the expression of the lipase fusion protein. As a control host cells *E. coli* BL21(DE3) and *E. coli* BL21(DE3)pAT-LipBc without addition of IPTG were cultivated and outer membranes were prepared and analyzed identically (Figure 2A, lanes 1 and 2). Inducing the protein expression of *E. coli* BL21(DE3) pAT-LipBc resulted in expression of the lipase fusion protein with a size of ~82 kDa (Figure 2A, lane 3). A lipase specific antibody was available, so the correct surface exposure of lipase could be evaluated by fluorescence-activated cell sorting (FACS). Since antibodies are too large to cross the outer membrane, they can only bind on surface exposed structures [36]. Therefore, cells expressing a passenger protein on their surface which is then marked by fluorescently labeled antibodies can easily be detected by FACS and will thereby cause an increase in fluorescence values compared to cells without such surface displayed protein. To identify effects caused by unspecific binding, the native host strain *E. coli* BL21(DE3) and another autodisplay strain displaying a different enzyme (NADH oxidase) on its surface (*E. coli* BL21(DE3) pAT-NOx) were used as controls. It turned out that the sample containing the lipase expressing cells showed a tenfold increase in mean fluorescence intensity values (Figure 3C) compared to the samples used as controls which showed no increased fluorescence signal (Figure 3A and B). The lipase antibody thus effectively bound the enzyme but did not show unspecific binding effects. Therefore the lipase expressed via autodisplay can be regarded as surface exposed. Interestingly, like Yang et al. [19] were already able to demonstrate, antibody labeling of the surface exposed lipase does not require the involvement of its chaperone foldase.

### Construction of the plasmid for autodisplay of foldase

According to Quyen *et al*. [12] the gene for foldase contains a possible N-terminal 70 aa membrane anchor. This structure is not required for the chaperone function of foldase, but may interfere with correct surface expression via autodisplay. Therefore foldase also was amplified from plasmid pHES8, which encodes the whole lipase operon [12], deleting the first 210 bp encoding this particular anchor structure. PCR primers, designed using the deposited sequence of the whole *B. cepacia lipase* [GenBank:FJ638612] added an XhoI site at the 5′-end and a KpnI site at the 3′-end of the foldase gene, analogously as described for the construction of plasmid pAT-LipBc. The derived fragment was ligated into autodisplay vector pBL001, digested with XhoI and KpnI before. Vector pBL001 is a pCOLA Duet^TM^ derivative, encoding the domains needed for autodisplay. Vector pBL001 furthermore provides a kanamycin resistance. Insertion of the foldase gene into pBL001 resulted in plasmid pAT-FoldBc encoding an in frame fusion of the autodisplay domains with foldase as a passenger (Figure 1C).

### Surface display of foldase

*E. coli* BL21 (DE3) pAT-FoldBc cells were grown to midlog phase and autotransporter fusion protein expression (FoldBc-FP) was induced by adding 1 mM IPTG to the fermentation broth and incubating the culture for another hour. After preparation of the outer membrane fraction, obtained protein samples were subjected to SDS-PAGE. As can be seen in Figure 2B, induction of protein expression resulted in the appearance of a protein band with an apparent molecular mass of around 80 kDa (Figure 2B, lane 2), which is in good accordance with the calculated molecular mass of 78.5 kDa for FoldBc-FP. The SDS-analysis revealed the location of the autotransporter fusion protein in the outer membrane protein fraction. The investigation of surface exposure via FACS was not possible for foldase, since there was no specific antibody against foldase available. Therefore, to elucidate if the passenger domain of FoldBc-FP is truly surface exposed and not directed to the periplasm, the accessibility of the fusion protein for proteases was tested. Since proteases are too large to pass the outer membrane, only surface exposed proteins will be degraded. In order to perform this degradation test whole cells of *E. coli* BL21(DE3) pAT-FoldBc were incubated with different concentrations of proteinase K. This treatment resulted in degradation of FoldBc-FP (Figure 2B, lanes 3 and 4). To demonstrate the integrity of the outer membrane during protease treatment, outer membrane protein A (OmpA) can be used as a reporter. The C-terminal part of OmpA directs into the periplasmic space while the N-terminal part builds a compact β-barrel structure inside the outer membrane [37]. A digestion of OmpA therefore can only occur from the periplasmic side, indicating that the outer membrane lost its integrity to enable the access for proteases into the periplasm. Thus, the fact, that the performed protease accessibility test led to a strong decrease of FoldBc-FP intensity (Figure 2B, lanes 3 and 4), without affecting OmpA intensity, provides strong evidence for the surface exposure of FoldBc-FP.

### Coexpression of both LipBc-FP and FoldBc-FP

**Figure 4:**
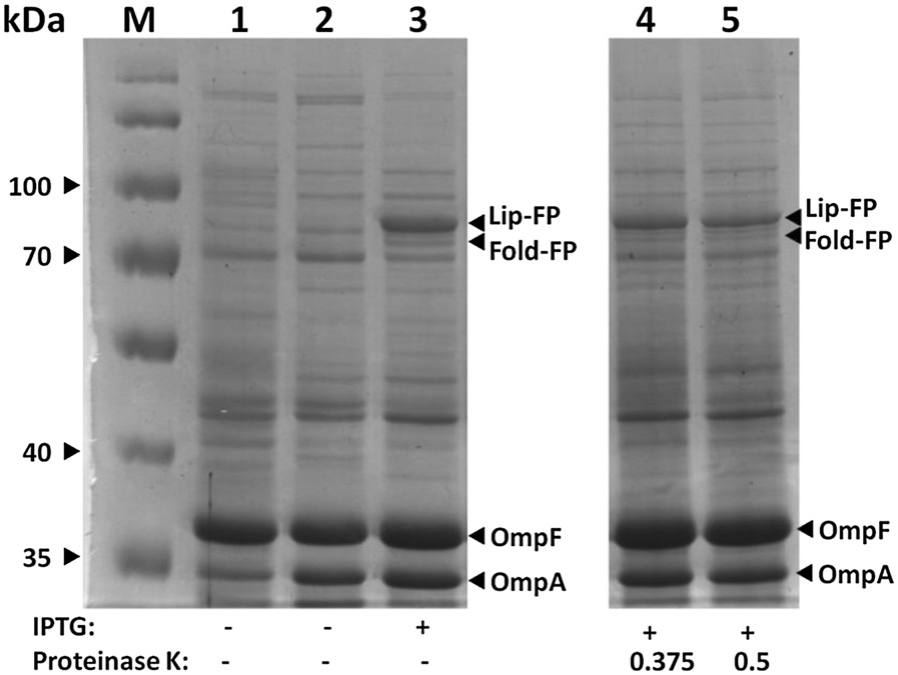
**Coexpression and surface display of both lipase and foldase fusion protein.** SDS-PAGE of membrane preparations of *E. coli* BL21(DE3)pAT-LiFoBc, coexpressing the lipase and foldase fusion protein. Molecular weight markers are indicated on the left hand side. Lane 1 shows a membrane preparation of *E. coli* BL21(DE3), used as a control. Lanes 2-5 show outer membrane preparations of *E. coli* BL21(DE3)pAT-LiFoBc. M: protein marker; IPTG: protein expression was induced by adding IPTG (final concentration: 1 mM); Proteinase K: whole cells were treated with Proteinase K, concentrations are given in mg mL^-1^; OmpA/OmpF: native *E. coli* outer membrane proteins. The foldase and lipase fusion proteins are indicated by black arrows.

Activity of the lipase from *Burkholderia cepacia* is dependent on the presence of foldase, a specific chaperone, enabling the correct folding of the lipase [1,12]. Since *E. coli* BL21(DE3) pAT-LipBc cells showed no lipase activity at all (data not shown), co-expression of pAT-LipBc together with pAT-FoldBc in one host was conducted. To bring both plasmids into one *E. coli* expression strain, plasmid pAT-FoldBc was transformed into electrocompetent cells of *E. coli* BL21(DE3)pAT-LipBc. Since both plasmids encode for different antibiotic resistances, transformants harboring pAT-LipBc and pAT-FoldBc could be identified by using selection media containing carbenicillin as well as kanamycin. The obtained strain was named *E. coli* BL21(DE3)pAT-LiFoBc. Cells co-expressing both LipBc-FP and FoldBc-FP were also investigated for correct surface display of both autotransporter fusion proteins. Therefore co-expression of both proteins was induced and cells were treated with proteinase K as described above in order to determine the accessibility of lipase and foldase fusion protein on the surface of one *E. coli* strain for externally added proteases. Proteinase K treatment resulted in digestion of both fusion proteins (Figure 4, lanes 4 and 5). The decrease in intensity of the fusion protein bands in comparison to the non-treated sample (Figure 4, lane 3) indicated their surface exposure. Additionally, the constant intensity of OmpA protein band indicates, that the cell integrity was sustained throughout this experiment.

### Lipase Activity of whole cells co-expressing LipBc-FP and FoldBc-FP

**Figure 5:**
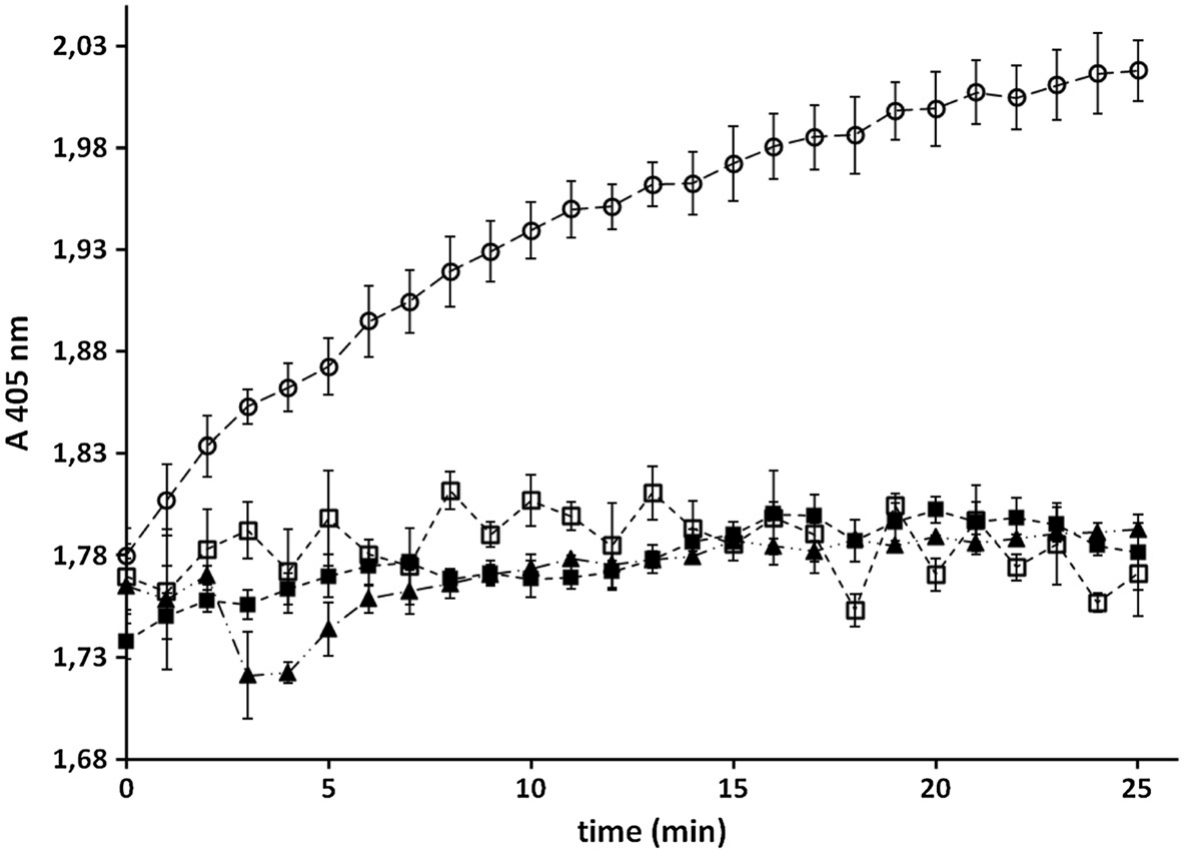
**Enzyme activity of whole cells displaying lipase and foldase.***p*-nitrophenyl palmitate was used as substrate and the increase of absorption at 405 nm was observed photometrically. The assay was performed in potassium phosphate buffer pH 7.4 at a constant temperature of 25°C. The increase in absorption is caused by the nitrophenylate anion after lipolytic cleavage of the ester bond conducted by the surface displayed lipase. **◯** = *E. coli* BL21(DE3)pAT-LiFoBc coexpressing both lipase and foldase ▲ = *E. coli* BL21(DE3) pAT-LipBc and *E. coli* UT5600(DE3) pAT-FoldBc mixed and preincubated for one hour ■ = *E. coli* BL21(DE3), host strain used as a control, ☐ = substrate solution in buffer. Mean values with standard deviations are shown, n = 3.

Lipases are known to split ester bonds and an established and easily performable assay to determine lipase activity is the lipolytic degradation of *p-*nitrophenyl palmitate (*p*-NPP) into *p*-nitrophenolate and palmitate. The nitrophenolate anion is colored yellow and its formation can be followed spectrophotometrically at 405 nm (ε_nitrophenol_ = 17,000 L mol^-1^ cm^-1^). To determine the lipase activity of whole cells, *E. coli* BL21(DE3)pAT-LiFoBc was cultivated and protein expression was induced as described above. As a control the host strain *E. coli* BL21(DE3) without a plasmid was cultivated analogously. Cells were then washed twice and resuspended to an OD_578_ of 10 in potassium phosphate buffer (25 mM, pH 7.4). For enzymatic conversion 20 μl of these cells were added to 180 μl of a 0.29 mM *p*-NPP solution in phosphate buffer (25 mM, pH 7.4) resulting in a final substrate concentration of 0.26 mM and a final OD_578_ =1. The assay was performed in in a 96-well plate and the kinetics of lipase reaction was measured as the increase in absorption at 405 nm for 25 min in a microplate reader at a constant temperature of 25°C. An increase of absorption values could only be measured in the samples containing *E. coli* BL21(DE3) pAT-LiFoBc (Figure 5). The host strain *E. coli* BL21(DE3) showed no significant increase in absorption at all. By using the initial enzyme reaction at min 1-4, the extinction coefficient of *p*-NPP and a pathway of 0,52 cm for a 200 μl reaction volume in the microplate reader, an activity of 2.73 mU/ml could be calculated for *E. coli* BL21(DE3) pAT-LiFoBc cells co-expressing lipase and foldase, applied at an OD_578_ of 1.

In addition, we investigated whether mixing the cells displaying only the lipase with cells displaying only the foldase could lead to whole cell lipase activity. This approach was somehow similar to that of Wilhelm et al. [18], who mixed cells displaying foldase with a denatured lipase and ended up with lipase activity. In our investigation, for the combination of both types of cells, *E. coli* BL21(DE3) pAT-LipBc and *E. coli* BL21(DE3) pAT-FoldBc were cultivated separately and protein expression was induced as described above. Each type of cells was washed and suspended to an OD_578_ of 10 as described before. Subsequently *E. coli* BL21(DE3) pAT-LipBc and *E. coli* BL21(DE3) pAT-FoldBc were mixed in a ratio of 1:1. Half of the sample was incubated for one hour, the other half was incubated for 24 hours at 20°C with vigorous shaking (200 rpm) to avoid sedimentation. After the incubation enzymatic activity was determined as described for the cells co-expressing lipase and foldase. However, mixing the cells displaying the foldase with cells displaying the lipase did not yield any activity at all, neither after 1 h nor after 24 h. This is to indicate that the surface displayed lipase needs to be co-expressed with its chaperone foldase on the surface of a single cell to gain its enzymatic activity.

### Lipase activity of outer membrane preparations from *E. Coli* BL21(DE3) pAT-LiFoBc

**Figure 6:**
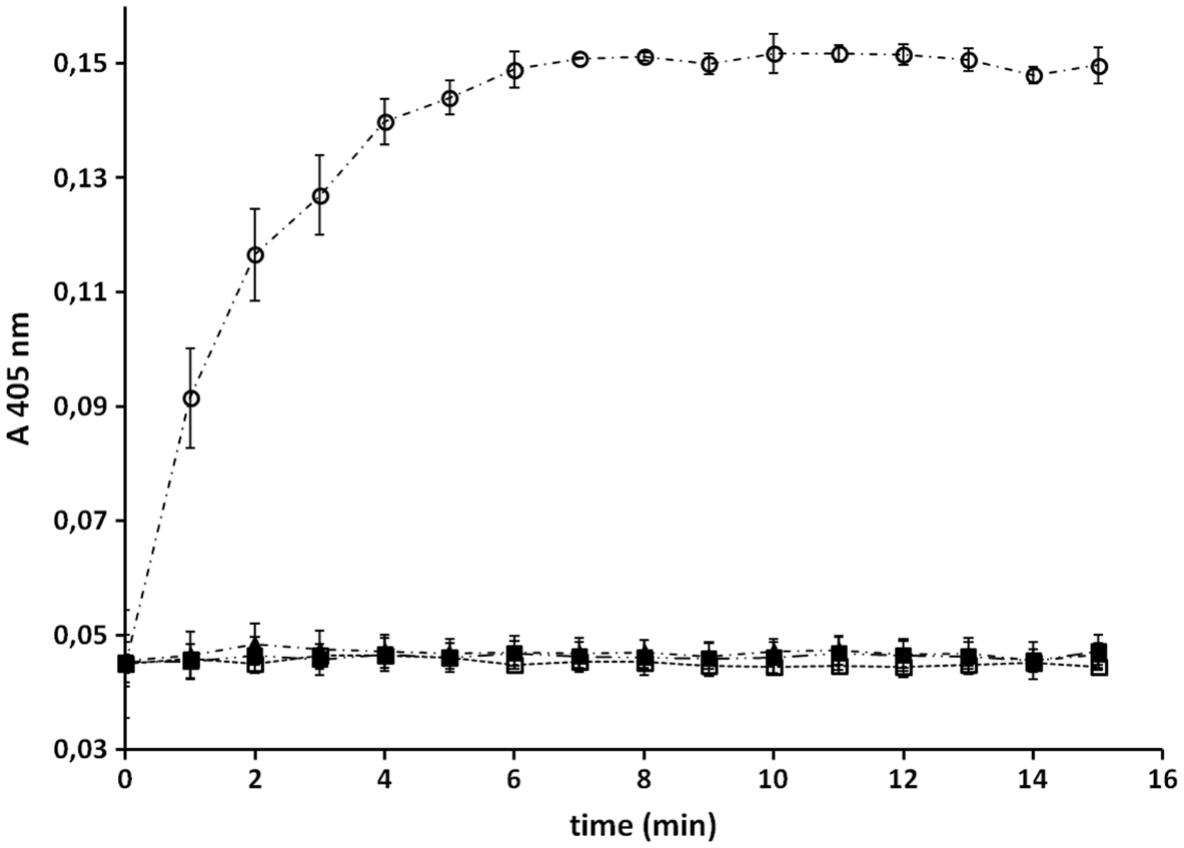
**Enzyme activity of outer membrane preparations obtained from cells displaying lipase and foldase.** Outer membranes were prepared as described in materials and methods and then applied to an assay with *p*-nitrophenyl palmitate as substrate and in which the increase of absorption at 405 nm was observed photometrically. The assay was performed in potassium phosphate buffer pH 7.4 at a constant temperature of 25°C. The increase in absorption is caused by the nitrophenylate anion after lipolytic cleavage of the ester bond conducted by the surface displayed lipase. **◯** = outer membrane preparations of *E. coli* BL21(DE3)pAT-LiFoBc coexpressing both lipase and foldase, ■ = outer membrane preparations of *E. coli* BL21(DE3) used as control, ☐ = substrate solution in buffer. Mean values with standard deviations are shown, n = 3.

In order to apply not only whole cells but membrane preparations for further washing experiments, the described enzyme assay was carried out with samples of membrane preparations as well. Membrane preparations were derived from *E. coli* BL21(DE3) pAT-LiFoBc and from previously combined *E. coli* BL21(DE3) pAT-LipBc and *E. coli* BL21(DE3) pAT-FoldBc. To obtain the outer membrane proteins, the preparation was performed according to a protocol described by Schultheiss et al [35] (see materials and methods). After the washing steps, outer membrane proteins were suspended in 1 mL of 25 mM phosphate buffer (pH 7.4). 20 μL of a 200 μL assay sample volume was composed of the membrane protein suspension which was corresponding to an amount of cells with a final OD_578_ of 2. As we anticipated that outer membrane preparation could lead to a loss in proteins and/or enzymatic activity, the amount of outer membrane proteins were taken from double the amount of cells assayed in the whole cell activity determination. The photometrical assays were then carried out at 25°C according to the same protocol as was used for whole cells. Only membrane protein preparations of the strain co-expressing enzyme and chaperone (*E. coli* BL21(DE3) pAT-LiFoBc) showed lipase activity (Figure 6). From the linear part of the curve in Figure 6 the enzymatic activity was determined to be 4.01 mU/ml, whereas membrane preparations of native *E. coli* BL21(DE3) cells as well as those of the pre-incubated cell mixture of *E. coli* BL21(DE3) pAT-LipBc and *E. coli* BL21(DE3) pAT-Fold-Bc showed no lipase activity at all (Figure 6). The determined activity for the membrane preparation from the cells coexpressing lipase and foldase on the surface was only by a factor of 1.5 higher than the activity of whole cells when applied in the same assay. But as described above the outer membrane proteins from double the amount of cells were applied, referring to the corresponding OD_578_.This indicates a loss of function or even a loss of the lipase and/or foldase during the preparation protocol, but could also been due to a general loss in cellular material during the centrifugation step. Nevertheless the enzyme, co-expressed with its chaperone, showed activity not only on the surface of *E. coli* cells but also in preparations of outer membrane proteins derived thereof.

### Application of cells and membrane preparations in a standardized laundry test

**Figure 7:**
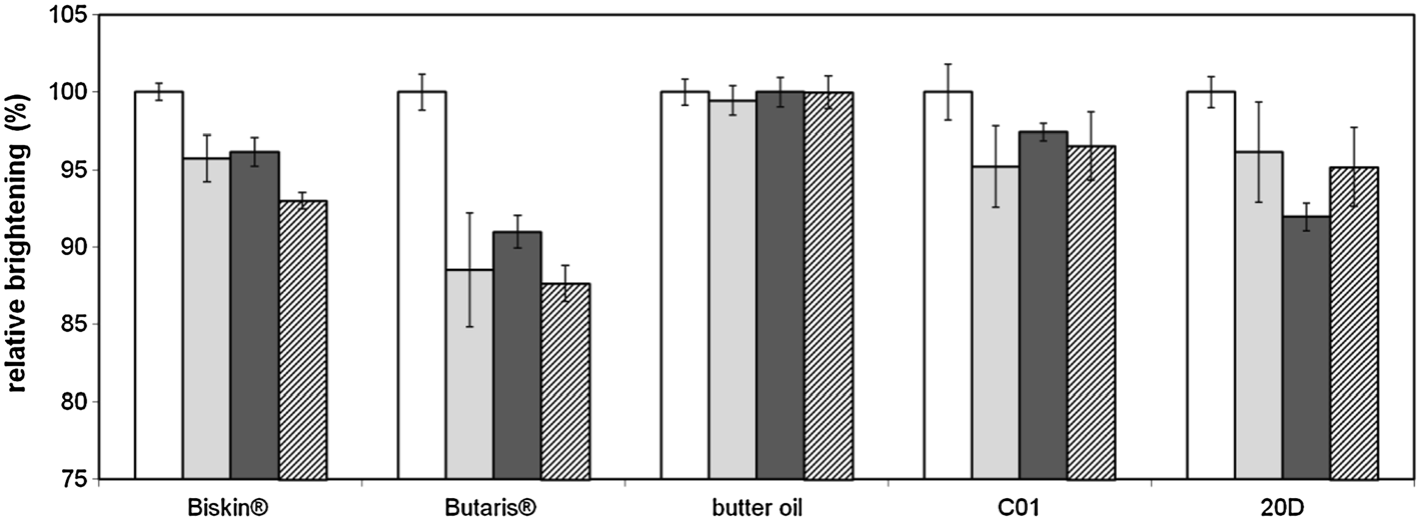
**Laundry cleaning with different lipase samples in the Linitest plus testing system.** The brightening effect caused by soluble lipase, the lipase-whole cell biocatalyst and the membrane preparation, respectively are shown in per cent relatively to the brightening effect caused by the detergent lipase. Average values determined from three measurement points and standard deviations are depicted. White bars: detergent lipase, light grey bars: soluble lipase from *B. cepacia*, dark grey bars: the herein described lipase-whole cell biocatalyst, shaded bars: membrane preparations thereof.

One major aim of this study was the application of an autodisplay whole-cell biocatalyst in a real-life laundry process. Therefore the lipolytic capability of *E. coli* BL21(DE3) pAT-LiFoBc and membrane preparations thereof was determined in a standardized test imitating a conventional machine washing process. During this test, cells and membrane fractions were compared to soluble, reconstituted lipase from *B. cepacia* and Lipex® a lipase preparation, which is already applied in washing agents. It turned out, that there was no significant difference in lipase activity between the soluble enzyme from *B. cepacia*, the lipase-whole cell biocatalyst and membrane preparations thereof. These results indicate that the lipase-whole cell biocatalyst and its membrane preparation endured the mechanically demanding procedure (test cloth and steel balls within the washing vessel, 40°C, 45 rpm) yielding up to 100% of the lipolytic performance given as relative brightening effect of Lipex® against Butaris® (Figure 7). Lipolytic performance against the other tested fat and grease spots moved in the range of 90-95% relative activity compared to Lipex®. The membrane stabilization of lipase by autodisplay therefore obviously revealed no significant improvement in efficiency compared to soluble lipase within this test. Nevertheless, the low differentiation values between the tested enzyme preparations and the relatively high standard deviations are presumably due to the small scale testing which was applied here. Since this might be a statistical problem, a more exact determination of differences between the several preparations of lipase may be overcome by an enlargement of the test set up and the application of a larger number of samples. Furthermore a better differentiation may be obtained by a more precise determination of the exact number of enzymes on a single whole-cell-biocatalyst and thus the number of enzymes applied in one sample, which is possible by flow cytometry, for example. Nonetheless it needs to be considered, that this was the first time, whole cells with a surface displayed lipase and membrane preparations thereof were subjected to a process like this.

## Discussion

Since ecologically friendly housekeeping processes become more and more important for a broad public and within a steadily growing biotechnological industry the need for cost efficient and easy accessible lipase preparations increases. By means of Autodisplay a new method to make the challenging lipase from *B. cepacia* easily available was developed: Within this study we were for the first time able to use Autodisplay for the co-expression of two different proteins, which need to interact with each other, a lipase and its implicitly required chaperone, foldase. By co-expression of both these proteins on the surface of one single *E. coli* cell we obtained a functional lipase-whole cell biocatalyst. Simply combining two cell types, each displaying one of the proteins, either lipase or foldase was not sufficient to create a functional whole cell biocatalyst. This indicates that the interaction between lipase and foldase can only take place if they are expressed on the surface of a single cell. Therefore, it can be assumed that a certain vicinity of lipase and foldase is needed for the process of folding supported by the chaperone. The novelty of the present investigation is, that the lipase and its specific foldase were expressed separately and both proteins interacted spontaneously and self driven, finally yielding an enzymatically active lipase at the cell surface of *E. coli*. In this respect the study goes beyond the aims of Wilhelm et al., [18], which displayed a foldase on the surface of *E. coli* and added the corresponding lipase as a purified protein subsequently and it goes an important step further than the work of Yang et al. [19] who obtained the surface display of an active lipase after co-expression with foldase in a single fusion protein. Our report is the first time description of the separate expression and surface display of two enzymes that finally interacted with each other in order to obtain an enzymatic activity. It paves the way for the surface display of other multiprotein or multienzyme complexes by a similar strategy, which was to the best of our knowledge up to now not taken into consideration. Our data show, that this interaction and the anchorage within the *E. coli* outer membrane deliver a biocatalyst stable enough to endure even a stressing and mechanically demanding procedure like the standardized laundry tests which had been conducted here. The whole cell biocatalyst and the membrane preparations yielded an activity in the same order of magnitude to the purified enzyme and a standard lipase formulation already used in detergents (Lipex®). Taken the activity 0f 4.01 mU/ml at an OD_578_ = 1 as an example, the whole cell lipase/foldase biocatalyst described here would reduce the costs in a 30 qm fermenter to 35% of those required for the purified enzyme to get the same amount of product, taken into consideration fermentation, purification and stabilization of the catalysts, as well as the necessary raw materials (G. Festel, unpublished). But it would be also possible to gain an even higher enzymatic activity by *E. coli* BL21(DE3) pAT-LiFoBc which exceeds the activity of purified and reconstituted *B. cepacia* lipase and the detergent lipase by further optimization of the culturing conditions and culture medium for instance. Moreover directed evolution approaches or site-directed mutagenesis could be applied in order to gain higher lipase activities finally.

## Conclusion

Autodisplay offers once more a convenient alternative to obtain a functional biocatalyst without precedent laborious purifying steps and in the special case of *B. cepacia* lipase and its chaperone foldase without a strongly required reconstitution protocol. The successful removal of fat or grease spots respectively during standard washing procedures was possible by simply applying surface engineered cells and *E. coli* outer membrane preparations containing active surface displayed lipase. Working with a cell-free preparation which achieves the same activities like the whole cell biocatalyst is therefore also feasible. These results give an outlook of possible applications for enzymes utilized by Autodisplay beyond laboratory scale testing.

## Methods

### Bacterial strains, plasmids and culture conditions

*Escherichia coli* strains UT5600(DE3) [Fˉ, *ara*-14, *leu*B6, *sec*A6, *lac*Y1, *pro*C14, *tsx*-67, Δ(*omp*T-fepC)266, *ent*A403, *trp*E38, *rfb*D1, *rps*L109(Str^r^), *xyl*-5, *mtl*-1, *thi*-1, λ(DE3)] and *E. coli* BL21(DE3) [B, Fˉ, *dcm*, *omp*T, *lon*, *hsdS*(rBˉ mBˉ), *gal*, λ(DE3)] were used for the expression of autotransporter fusion proteins. *E. coli* TOP10 (F- *mcr*A Δ(*mrrhsd*RMS-*mcr*BC) ϕ80*lac*ZDM15 Δ*lac*X74 *deo*R *rec*A1 *ara*D139 Δ(*ara-leu*) 7697 *gal*U *gal*K *rps*L (Str^R^) *end*A1 *nup*G) and the vector pCR®4-TOPO® were used for subcloning of polymerase chain reaction (PCR) products, using the TOPO-TA cloning kit (Invitrogen, Carlsbad, CA, USA). Site directed mutagenesis of the restriction sites for XhoI and KpnI inside the genes of interest was performed using the QuikChange Site Directed Mutagenesis Kit (Stratagene, Santa Clara, CA, USA) and appropriate mutagenesis primers. Construction of plasmid pCD003 which encodes the AIDA-I autotransporter has been described elsewhere [25]. Plasmid pBL001 is a pCOLA-Duet^TM^-1–derivative. The second MCS had been removed and the autotransporter cassette was inserted using NcoI and BlpI restriction sites. Plasmid pHES8, encoding the lipase and foldase from *Burkholderia cepacia*, is a derivative of pHES12, which has been described by Quyen et al. [12]. Bacteria were routinely grown at 37°C in Lysogeny broth (LB) containing carbenicillin (100 mg L^-1^) or kanamycin (30 mg L^-1^) or both antibiotics, respectively. For co-expression of both, lipase and foldase, a culture from strain *E. coli* BL21(DE3) pAT-LipBc, already containing the plasmid encoding for lipase-autotransporter fusion protein, was prepared to obtain electrocompetent cells according to a modified protocol from Sambrook et al. [38]. Plasmid pAT-FoldBc was then transformed into an aliquot of these cells by electroporation resulting in strain BL21(DE3)pAT-LiFoBc which contains both plasmids.

### Recombinant DNA techniques

For construction of plasmid pAT-LipBc, which contains the gene encoding LipBc-FP, the lipase gene was amplified by PCR. Plasmid pHES8 served as a template for primers EK009 (CGCTCGAGGCGAGCGCGCCCGCCGAC) and EK010 (GGTACCCACGCCCGCGAGCTTCAGCCG). To facilitate cloning of the lipase-PCR fragment into the autotransporter cassette, a XhoI restriction site was added to the 5′-end and a KpnI restriction site was added to the 3′-end via PCR. For construction of plasmid pAT-FoldBc, containing the gene which encodes for FoldBc-FP, the foldase gene was amplified by PCR, again using pHES8 as a template for primers CD004 (CTCGAGCCGTCGTCGCTGGCCGGCTCC) and CD005 (GGTACCCTGCGCGCTGCCCGCGCCGCG). 5′-XhoI and 3′-KpnI-restriciton sites were attached to the PCR fragment analogously. Both PCR products were each inserted into vector pCR®4-TOPO® and first brought to site directed mutagenesis according to the protocols delivered by Stratagene to remove unwanted restriction sites within the genes of interest. Mutated plasmids were then restricted with XhoI and KpnI. The restriction fragment containing the lipase gene was ligated into pET-derivative pCD003 [25] restricted with the same enzymes. The restriction fragment containing the *foldase* gene was ligated into pCOLA-Duet^TM^-1–derivative pBL001 restricted with the same enzymes before. Both ligation steps yielded an in frame fusion of lipase or foldase respectively, with the autotransporter domains under the control of a T7/lac promoter. Plasmid DNA preparation, restriction digestion, ligation, DNA electrophoresis and transformation were performed according to standard protocols [38]. Gel extraction of digested fragments was performed using a gel extraction kit from Qiagen (Hilden, Germany).

### Outer membrane protein preparation

*E. coli* cells were grown overnight and 1 ml of the culture was used to inoculate LB medium (40 ml). Cells were cultured at 37°C with vigorous shaking (200 rpm) for about 2 hours until an OD_578_ of 0.5 was reached. The culture was separated into two aliquots and protein expression was induced by adding IPTG at a final concentration of 1 mm to one of the aliquots. Cultures then were incubated at 30°C and shaking (200 rpm) for one hour. Induction was stopped by incubating the cells on ice for 15 min. After harvesting and washing of the cells with Tris-HCl (0.2 m, pH 8), differential cell fractionation was performed according to the method of Hantke [34] as modified by Schultheiss *et al*. [35] In detail, cell lysis was obtained by adding lysozyme (0.04 mg/mL end concentration) in the presence of 10 mM saccharose and 1 μM EDTA in a final volume of 1.5 mL of Tris-HCl (0.2 M, pH 8) and incubation for 10 min at room temperature. Subsequently aprotinin (10 μg/mL), phenylmethylsulfonyl fluoride (PMSF) (0.5 mM), as well as 5 mL of extraction buffer (50 mM Tris-HCl pH8.0, 10 mM MgCl_2_, 2% Triton × 100) and DNAseI (10 μg/mL) were added. After incubation on ice for 30 min the samples were centrifuged (2,460 g, 5 min, 4.0°C) to remove intact bacteria and large cell debris. The supernatants representing the clarified bacterial lysate were retained and centrifuged at higher speed (38,700 × g, 30 min, 4.0°C) in order to obtain the membrane protein fraction. The resulting supernatant, containing soluble cytoplasmic and periplasmic proteins, was completely aspirated. The pellet was suspended in 10 ml phosphate-buffered saline (PBS) plus 1% Sarcosyl (*N-*lauryl sarcosinate, sodium salt) and centrifuged again (38,700 × g, 60 min, 4°C). The supernatant after this step contained the sarcosyl-soluble cytoplasmic membrane proteins and was completely aspirated. The sediment representing the outer membrane protein fraction was washed twice with 10 ml of water and dissolved in 30 μl water for SDS-PAGE or an adequate volume of 25 mM potassium phosphate buffer pH 7,4 for activity determination. For whole cell protease treatment, *E. coli* cells were harvested, washed and resuspended in 1 ml Tris-HCl (0.2 m, pH 8). Proteinase K was added to final concentrations between 0.2 mg mL^-1^ and 0.5 mg mL^-1^ and cells were incubated for 1 hour at 37°C. Digestion was stopped by washing the cells twice with Tris-HCl (0.2 M, pH 8) containing 10% fetal calf serum (FCS) and outer membrane proteins were prepared as described above.

For outer membrane proteins that were applied for activity assays, cells were not treated with Proteinase K.

### SDS-PAGE

Outer membrane isolates were diluted (1:1.5) with sample buffer (100 mm Tris/HCl (pH 6.8) containing 4% SDS, 0.2% bromophenol blue, 200 mm dithiothreitol and 20% glycerol), boiled for 10 minutes and analyzed on 10% polyacrylamid gels. Proteins were stained with Coomassie brilliant blue (R250). To correlate molecular masses of protein bands of interest, a molecular weight standard was used (PageRuler unstained, Fermentas, Burlington, Canada).

### Flow cytometer analysis

*E. coli* BL21(DE3) pAT-LipBc cells were grown and expression of lipase fusion protein was induced as described above by adding IPTG to a final concentration of 1 mM and incubating the cells for another hour at 30°C under shaking (200 rpm). Cells were harvested by centrifugation (2400 g, 2 min, 4°C, Mikro200R, Hettich, Tuttlingen, Germany) and washed twice with filter sterilized (0.2 μm pore size, ethylethersulfone membrane) phosphate buffered saline (PBS, pH 7.4) before suspending to a final OD_578_ of 0.25/mL for further experiments. 100 μl of these cells were again centrifuged and resuspended in 500 μL PBS (pH 7.4) containing 3% bovine serum albumin (BSA, filter sterilized) and incubated for 10 min at room temperature. After centrifuging the cells for 60 sec with 17,000 g (Mikro200R, Hettich, Tuttlingen, Germany), the obtained cell pellet was suspended with 100 μL of rabbit anti lipase antibody (diluted 1:50 in PBS (pH 7.4) + 3% BSA, filter sterilized) and incubated for another 30 min at room temperature. Subsequently cells were washed twice with 500 μL of PBS (pH 7.4) + 3% BSA. Cell pellets were resuspended in 100 μL of secondary antibody solution (goat-anti-rabbit, Dylight^TM^ 633, Thermo Scientific, diluted 1:25 in PBS (pH 7.4) +3% BSA) and incubated for 30 min in the dark at room temperature. After washing twice in 500 μL of PBS (pH 7.4) the cell pellet was finally suspended in 1.5 mL of PBS (pH 7.4, filter sterilized to avoid external particles). The samples were analyzed using a flow cytometer (Cyflow Space, Partec, Münster, Germany) at an excitation wavelength of 647 nm.

### Lipase activity assay

Photometrical Assays to determine lipolytic activity of the lipase-whole cell biocatalyst were performed according to a modified protocol by Winkler and Stuckmann [39] with *p*-nitrophenylpalmitate (*p*-NPP) as substrate. For this purpose cells were routinely cultivated in LB medium until an optical density at 578 nm (OD^578^) of 1.0 was reached. Induction of protein expression was started by adding IPTG at a final concentration of 1 mM and incubating the cells another hour at 30°C and 200 rpm. Cells were then harvested by centrifugation and washed twice in potassium phosphate buffer, 25 mM, pH 7.4, and stored in the same buffer at 4°C in an OD_578_ = 10 until used for assays. In case of mixing different types of cells, they were used in a 1:1 ratio at OD_578_ =10 and incubated at 20°C on a rocking platform to avoid sedimentation For activity assays a stock solution of the substrate *p*-NPP was prepared in ethanol to a final concentration of 7.9 mM) and finally diluted in potassium phosphate buffer, 25 mM, pH 7.4 under constant stirring to a working concentration of 0.29 mM. This working solution was prepared freshly, kept at 25°C for one hour before its application and was not used when a visible turbidity or a yellow coloring occurred. Activity measurement was started by adding 180 μl of this working solution to 20 μl of cells with an OD_578_ = 10. This yielded a final substrate concentration of 0.26 mM and a final OD_578_ = 1 of the cells in the assay. The lipolytic production of yellow colored nitrophenylate at 25°C was measured at 405 nm in a 96 well plate using a microplate reader (Mithras LB940, Berthold, Bad Wildbach, Germany). The linear increase in absorption was used to calculate the enzymatic activity according to the law of Lambert and Beer (ε_nitrophenol_ = 17,000 L mol^-1^ cm^-1^, d = 0,52 cm path length correction for the layer thickness of 200 μL assay volume in one well). One unit was defined as the amount of enzyme which caused the release of 1 μmol of *p*-NPP per minute [12]. For measuring the lipolytic activity of outer membrane protein preparation we proceeded similarly, with the exception, that the 20 μl which were added to the 180 μl assay buffer with the substrate were derived from an amount of cells corresponding to an OD_578_ = 2. For these activity measurements, absorption values at 405 nm obtained with outer membrane preparations in potassium phosphate buffer without the addition of *p*-NPP were used for blank correction.

### Laundry tests with lipase-whole cell biocatalyst/ *E. coli* BL21(DE3) pAT-LipBc

The capability of lipase was tested on five different, standardized, lipase sensitive staining. The staining contained either Biskin® (Peter Kölln KGaA, Elmshorn, Germany), Butaris® (DFF Dairy Fine Food GmbH, Ratzeburg, Germany) or butter oil or a mixture of soot and mineral oil (C01, Center for Test Materials, Vlaardingen, The Netherlands) and a mixture of cutaneous sebum and pigment (20D, wfk Testgewebe GmbH, Krefeld, Germany) respectively. Tested lipases were a) a standard lipase preparation which is already used for washing purposes, b) soluble lipase from *B. cepacia*, c) the herein described lipase-whole cell biocatalyst and d) a membrane preparation thereof. To allow comparability, all lipases were applied in the same amounts, related to enzymatic activity. The washing process was carried out in a Linitest Plus (Atlas, Rock Hill, SC, USA), which represents the minituarized form of a standard machine washing process. The washing solution was prepared with 3.53 g of an enzyme free liquid detergent similar to a european premium detergent in water (16 °dH) buffered with 50 mM sodium phosphate pH 7.0. The washing process took place in a total volume of 170 mL at 40°C and 45 rpm for 60 minutes. To simulate the mechanism of a standard washing process, 10 steel balls were added and filled up with test cloth to a total amount of 14.3 g textile weight. Subsequently the test cloth was rinsed three times with deionized water and dried at room temperature in the dark. Color measurement of the staining was then carried out with a Minolta colorimeter (Konica-Minolta, München-Neuperlach, Germany), calibrated against producer’s standards, applying CIE L*a*b*, D65/10°/SCI settings. Each staining was measured three times and the average L* value was determined.

## Competing interests

The presented study arose from a larger project within the scope of a patent value fund. In this context we acknowledge the financial support provided by the Zyrus Beteiligungsgesellschaft mbH & Co. Patente I KG (Germany). Zyrus, the company that financially supported our study, submitted a patent application on parts of the study (autodisplay of an active lipase). EK and CD are working now for a company called “Autodisplay Biotech”.

## Authors’ contributions

EK carried out cloning of the lipase fusion protein, performed the coexpression of lipase and foldase, arranged and conducted all lipase activity tests in laboratory scale and drafted the main part of the manuscript. CD carried out cloning of the foldase fusion protein, participated in the coexpression studies and wrote minor parts of the manuscript. TW carried out the standardized laundry test for fatty stain removal. JJ conceived of the study, guided its design and coordination and wrote parts of the manuscript. All authors read and approved the final manuscript.

## References

[B1] Arpigny JL Jaeger KE Bacterial lipolytic enzymes: classification and properties Biochem J 1999 343 177 183 10.1042/0264-6021:3430177 10493927 PMC1220539

[B2] Reetz MT Lipases as practical biocatalysts Curr Opin Chem Biol 2002 6 145 150 10.1016/S1367-5931(02)00297-1 12038997

[B3] Tadashi E Mechanism of enantioselectivity of lipases and other synthetically useful hydrolases Curr Org Chem 2004 8 1009 1025 10.2174/1385272043370230

[B4] Jaeger KE Reetz MT Microbial lipases form versatile tools for biotechnology Trends Biotechnol 1998 16 396 403 10.1016/S0167-7799(98)01195-0 9744114

[B5] Sharma R Chisti Y Banerjee UC Production, purification, characterization, and applications of lipases Biotechnol Adv 2001 19 627 662 10.1016/S0734-9750(01)00086-6 14550014

[B6] Shu ZY Jiang H Lin RF Jiang YM Lin L Huang JZ Technical methods to improve yield, activity and stability in the development of microbial lipases J Mol Catal B-Enzym 2010 62 1 8 10.1016/j.molcatb.2009.09.003

[B7] Gupta R Gupta N Rathi P Bacterial lipases: an overview of production, purification and biochemical properties Appl Microbiol Biotechnol 2004 64 763 781 10.1007/s00253-004-1568-8 14966663

[B8] Gotor-Fernández V Brieva R Gotor V Lipases: useful biocatalysts for the preparation of pharmaceuticals J Mol Catal B-Enzym 2006 40 111 120 10.1016/j.molcatb.2006.02.010

[B9] Noureddini H Gao X Philkana RS Immobilized pseudomonas cepacia lipase for biodiesel fuel production from soybean oil Bioresour Technol 2005 96 769 777 10.1016/j.biortech.2004.05.029 15607189

[B10] Park DS Oh HW Heo SY Jeong WJ Shin DH Bae KS Park HY Characterization of an extracellular lipase in Burkholderia sp. HY-10 isolated from a longicorn beetle J Microbiol 2007 45 409 417 17978800

[B11] Quyen TD Vu CH Le GT Enhancing functional production of a chaperone-dependent lipase in Escherichia coli using the dual expression cassette plasmid Microb Cell Fact 2012 11 29 10.1186/1475-2859-11-29 22380513 PMC3359195

[B12] Quyen DT Schmidt-Dannert C Schmid RD High-level formation of active Pseudomonas cepacia lipase after heterologous expression of the encoding gene and its modified chaperone in Escherichia coli and rapid in vitro refolding Appl Environ Microb 1999 65 787 794 10.1128/aem.65.2.787-794.1999 PMC91096 9925617

[B13] Rutherford N Mourez M Surface display of proteins by Gram-negative bacterial autotransporters Microb Cell Fact 2006 5 22 10.1186/1475-2859-5-22 16787545 PMC1533851

[B14] Baek JH Han MJ Lee SH Lee SY Enhanced display of lipase on the escherichia coli cell surface, based on transcriptome analysis Appl Environ Microbiol 2010 76 971 973 10.1128/AEM.02463-09 19948866 PMC2812984

[B15] Jung HC Ko S Ju SJ Kim EJ Kim MK Pan JG Bacterial cell surface display of lipase and its randomly mutated library facilitates high-throughput screening of mutants showing higher specific activities J Mol Catal B-Enzym 2003 26 177 184 10.1016/j.molcatb.2003.05.007

[B16] Lee SH Choi JI Han MJ Choi JH Lee SY Display of lipase on the cell surface of Escherichia coli using OprF as an anchor and its application to enantioselective resolution in organic solvent Biotechnol Bioeng 2005 90 223 230 10.1002/bit.20399 15739170

[B17] Lee SH Choi JI Park SJ Lee SY Park BC Display of bacterial lipase on the Escherichia coli cell surface by using FadL as an anchoring motif and use of the enzyme in enantioselective biocatalysis Appl Environ Microbiol 2004 70 5074 5080 10.1128/AEM.70.9.5074-5080.2004 15345384 PMC520891

[B18] Wilhelm S Rosenau F Becker S Buest S Hausmann S Kolmar H Jaeger KE Functional cell-surface display of a lipase-specific chaperone Chembiochem 2007 8 55 60 10.1002/cbic.200600203 17173269

[B19] Yang TH Kwon MA Song JK Pan JG Rhee JS Functional display of pseudomonas and burkholderia lipases using a translocator domain of EstA autotransporter on the cell surface of escherichia coli J Biotechnol 2010 146 126 129 10.1016/j.jbiotec.2010.01.022 20138931

[B20] Kaessler A Olgen S Jose J Autodisplay of catalytically active human hyaluronidase hPH-20 and testing of enzyme inhibitors Eur J Pharm Sci 2011 42 138 147 10.1016/j.ejps.2010.11.004 21075205

[B21] Jose J Jahnig F Meyer TF Common structural features of IgA1 protease-like outer membrane protein autotransporters Mol Microbiol 1995 18 378 380 10.1111/j.1365-2958.1995.mmi_18020378.x 8709857

[B22] Maurer J Jose J Meyer TF Autodisplay: one-component system for efficient surface display and release of soluble recombinant proteins from *Escherichia coli* J Bacteriol 1997 179 794 804 9006035 10.1128/jb.179.3.794-804.1997 PMC178762

[B23] Jose J Bernhardt R Hannemann F Cellular surface display of dimeric Adx and whole cell P450-mediated steroid synthesis on *E. coli* J Biotechnol 2002 95 257 268 10.1016/S0168-1656(02)00030-5 12007866

[B24] Jose J von Schwichow S Autodisplay of active sorbitol dehydrogenase (SDH) yields a whole cell biocatalyst for the synthesis of rare sugars Chembiochem 2004 5 491 499 10.1002/cbic.200300774 15185373

[B25] Detzel C Maas R Jose J Autodisplay of nitrilase from alcaligenes faecalis in *E. Coli* yields a whole cell biocatalyst for the synthesis of enantiomerically pure (R)-mandelic acid ChemCatChem 2011 3 719-725 10.1002/cctc.201000382

[B26] Kranen E Steffan N Maas R Li S-M Jose J Development of a whole cell biocatalyst for the efficient prenylation of indole derivatives by autodisplay of the aromatic prenyltransferase FgaPT2 ChemCatChem 2011 3 1200 1207 10.1002/cctc.201000429

[B27] Schumacher SD Hannemann F Teese MG Bernhardt R Jose J Autodisplay of functional CYP106A2 in escherichia coli J Biotechnol 2012 161 104 112 10.1016/j.jbiotec.2012.02.018 22426093

[B28] Schumacher SD Jose J Expression of active human P450 3A4 on the cell surface of escherichia coli by autodisplay J Biotechnol 2012 161 113 120 10.1016/j.jbiotec.2012.01.031 22326629

[B29] Jose J Maas RM Teese MG Autodisplay of enzymes-molecular basis and perspectives J Biotechnol 2012 161 92 103 10.1016/j.jbiotec.2012.04.001 22569038

[B30] Jose J Meyer TF The autodisplay story, from discovery to biotechnical and biomedical applications Microbiol Mol Biol Rev 2007 71 600 619 10.1128/MMBR.00011-07 18063719 PMC2168652

[B31] Gawarzewski I Smits SH Schmitt L Jose J Structural comparison of the transport units of type V secretion systems Biol Chem 2013 394 1385 1398 23929883 10.1515/hsz-2013-0162

[B32] Economou A Following the leader: bacterial protein export through the Sec pathway Trends Microbiol 1999 7 315 320 10.1016/S0966-842X(99)01555-3 10431204

[B33] Dyrløv Bendtsen J Nielsen H von Heijne G Brunak S Improved prediction of signal peptides: SignalP 3.0 J Mol Biol 2004 340 783 795 10.1016/j.jmb.2004.05.028 15223320

[B34] Hantke K Regulation of ferric iron transport in *Escherichia coli* K12: isolation of a constitutive mutant Mol Gen Genet 1981 182 2 288 292 10.1007/BF00269672 7026976

[B35] Schultheiss E Paar C Schwab H Jose J Functional esterase surface display by the autotransporter pathway in *Escherichia coli* J Mol Catal B-Enzym 2002 18 89 97 10.1016/S1381-1177(02)00063-2

[B36] Jose J Handel S Monitoring the surface display of recombinant protiens by cysteine labeling ad flow cytometry ChemBioChem 2003 4 396 405 10.1002/cbic.200200530 12740811

[B37] Koebnik R Kramer L Membrane assembly of circularly permuted variants of the *E. coli* outer membrane protein OmpA J Mol Biol 1995 250 617 626 10.1006/jmbi.1995.0403 7623380

[B38] Sambrook J Fritsch EF Maniatis T Molecular Cloning. A Laboratory Manual 1989 Cold Spring Harbour, NY: Cold Spring Harbor Press

[B39] Winkler UK Stuckmann M Glycogen, hyaluronate, and some other polysaccharides greatly enhance the formation of exolipase by Serratia marcescens J Bacteriol 1979 138 663 670 222724 10.1128/jb.138.3.663-670.1979 PMC218088

